# Slipping through the Cracks: Rubber Plantation Is Unsuitable Breeding Habitat for Frogs in Xishuangbanna, China

**DOI:** 10.1371/journal.pone.0073688

**Published:** 2013-09-10

**Authors:** Jocelyn E. Behm, Xiaodong Yang, Jin Chen

**Affiliations:** 1 Xishuangbanna Tropical Botanic Garden, Chinese Academy of Sciences, Menglun, Yunnan, China; 2 Department of Zoology, University of Wisconsin, Madison, Wisconsin, United States of America; 3 Department of Ecological Science, Vrije Universiteit, Amsterdam, The Netherlands; Universität Zurich, Switzerland

## Abstract

Conversion of tropical forests into agriculture may present a serious risk to amphibian diversity if amphibians are not able to use agricultural areas as habitat. Recently, in Xishuangbanna Prefecture, Yunnan Province – a hotspot of frog diversity within China – two-thirds of the native tropical rainforests have been converted into rubber plantation agriculture. We conducted surveys and experiments to quantify habitat use for breeding and non-breeding life history activities of the native frog species in rainforest, rubber plantation and other human impacted sites. Rubber plantation sites had the lowest species richness in our non-breeding habitat surveys and no species used rubber plantation sites as breeding habitat. The absence of breeding was likely not due to intrinsic properties of the rubber plantation pools, as our experiments indicated that rubber plantation pools were suitable for tadpole growth and development. Rather, the absence of breeding in the rubber plantation was likely due to a misalignment of breeding and non-breeding habitat preferences. Analyses of our breeding surveys showed that percent canopy cover over pools was the strongest environmental variable influencing breeding site selection, with species exhibiting preferences for pools under both high and low canopy cover. Although rubber plantation pools had high canopy cover, the only species that bred in high canopy cover sites used the rainforest for both non-breeding and breeding activities, completing their entire life cycle in the rainforest. Conversely, the species that did use the rubber plantation for non-breeding habitat preferred to breed in low canopy sites, also avoiding breeding in the rubber plantation. Rubber plantations are likely an intermediate habitat type that ‘slips through the cracks’ of species habitat preferences and is thus avoided for breeding. In summary, unlike the rainforests they replaced, rubber plantations alone may not be able to support frog populations.

## Introduction

Amphibians are the most threatened vertebrate group worldwide, and habitat change is the leading cause of amphibian population declines [1–3]. Protected habitat is paramount for amphibian conservation, but the current amount of habitat in global protected areas is insufficient and most amphibian species must rely on unprotected areas for population persistence [4]. However, unprotected forests may not be a suitable alternative for conservation as they are rapidly being converted to agriculture particularly in tropical regions where amphibian diversity is high [5–7]. In a tropical landscape where agricultural patches are small and forest patches are large, amphibian population persistence is a function of how well they can disperse between forest patches through the intervening agricultural matrix [8]. As agriculture expands, however, and forest patches become smaller and more isolated, amphibian persistence may be a function of how well they can directly use agricultural patches as habitat [9,10]. Therefore accurate assessments of amphibian use of agricultural patches are imperative to predict a species’ future population persistence [4,11,12].

These issues are exemplified in the lowlands of tropical Xishuangbanna Prefecture, Yunnan Province, China, where an enormous diversity of organisms are being faced with an increasingly changing landscape. Part of the Indo-Burma biodiversity hotspot [13] Xishuangbanna is 0.2% of China’s land area yet contains over 14% of China’s reptiles and amphibians [14] and Xishuangbanna’s lowlands are a frog diversity hotspot within China [15,16]. In Xishuangbanna, 12% of the land is in protected areas, but these areas are isolated high elevation evergreen broad leaf forests, while lowland rainforests are largely unprotected [17]. Over the past 35 years, more than two-thirds of the native lowland rainforests have been replaced by monoculture plantations of Brazilian rubber trees ( *Hevea* *brasiliensis* ) [17]. This massive land conversion has caused the remaining lowland rainforests to experience significant fragmentation involving a decrease in mean patch size and an increase in interpatch distance, with 74% of the patches being less than 500 ha and on average 253 m apart [18]. Forest habitat patches in this region are small and isolated within vast stretches of rubber plantations.

The aim of our study is to determine whether native frog species use rubber plantations in Xishuangbanna as habitat. Although many studies have found frog species in tropical plantations during terrestrial habitat surveys [19–21] including rubber plantations in Brazil [22], it is necessary to consider both terrestrial and aquatic habitat to gain an accurate assessment of habitat use [23,24]. When suitable aquatic and terrestrial habitat patches are too far apart [23] or separated by inhospitable habitat [25], migration to breeding sites can result in mortality and eventually population declines. The majority of species in Xishuangbanna require aquatic breeding sites and breed in ephemeral rainwater pools. If they cannot successfully migrate through rubber plantations or directly use them as breeding habitat, populations may decline. Due to rubber plantation management methods, Xishuangbanna’s rubber plantations have a high density of ephemeral pools which may make them attractive to ephemeral pool breeders. Ephemeral pool-breeding amphibians can readily colonize new breeding sites in disturbed areas [26–28], but few studies have investigated the suitability of breeding habitat in plantations (but see 29).

Our approach was to explicitly consider both breeding (aquatic) and non-breeding (terrestrial) habitat use at the species level to understand the species’ habitat preferences within this modified landscape. We i) conducted surveys of species’ breeding and non-breeding habitat use; ii) identified environmental variables related to breeding habitat selection; and iii) assessed the quality of breeding habitat provided by rubber plantations.

## Methods

### Ethics Statement

All research described below was approved by the University of Wisconsin- Madison Animal Care and Use Committee (Protocol #L00393).

### Study System

Bordering Laos and Burma, Xishuangbanna prefecture is 19,700 km^2^, with elevations ranging from 550–1980 m. The ephemeral pool breeding frog species which were the focus of our study breed during the rainy season from May to October [14]. We conducted our surveys and experiments during the rainy seasons of 2008, 2009 and 2010 at the Xishuangbanna Tropical Botanic Garden (XTBG) (Longitude: 101^o^15'9.7″ E, Latitude: 21^o^55'44.63″ N). This 900 ha research institute is a microcosm of the larger Xishuangbanna landscape because it contains the major land cover types in Xishuangbanna including a 30-year-old, 195 ha working rubber plantation adjacent to one of Xishuangbanna’s few remaining patches of lowland rainforest. In general, most rubber plantations in Xishuangbanna are younger than XTBG’s [17]; therefore, XTBG’s rubber plantation represents a “best case-scenario” for frog colonization because it is a less-disturbed plantation adjacent to a source of frog species to colonize it.

### Breeding Survey

We conducted surveys to quantify breeding habitat use within the three major land-cover types in Xishuangbanna: rainforest, rubber plantation and impacted sites. Impacted is a general category we use for land-cover types that were not rubber plantation or rainforest and had some level of human impact, ranging from grassy fields to dirt roads. In 2008, we conducted preliminary surveys in each land-cover type to identify aquatic sites and learn the frog calls. Based on the location of these sites, in 2009 we established 2.8 km, 3.1 km, and 3.4 km routes for intensive surveys in rubber plantation, rainforest and impacted areas respectively. Survey routes consisted of aquatic sites connected by intervening stretches of terrestrial habitat along which we performed both breeding and non-breeding surveys. Although survey routes were not identical in length among the three areas, we opted to survey as many pools as possible in each area to obtain sufficient data to quantify habitat preferences.

The majority of our survey sites were small, temporary rainwater pools; however, we also surveyed a few more-permanent pools (ornamental concrete ponds at XTBG constructed within the last 30 years). In general very few pools in our survey, regardless of location, could be considered “natural”. Most were the result of depressions in the ground caused by human activities such as tire ruts and drainage ditches in impacted sites, and a dammed man-made stream in rainforest sites. Thus, these pools were a good representation of the types of aquatic sites available to frogs in the heavily impacted Xishuangbanna landscape.

We visited aquatic sites along our survey routes once per week at night to record calling activity and then revisited the same sites the following day to record environmental site variables and oviposition activity. We surveyed each route once per week for 11 consecutive weeks (June 8 – August 17) in the rainforest and impacted areas, and 9 consecutive weeks (June 22 – August 17) in the rubber plantation during the 2009 breeding season. At each breeding site, we listened for 5 minutes and recorded the total number of species calling. We then listened for an additional 2 minutes, and if no new species were heard the survey was over. If we did record new species, we added 2 minute segments until no new species were heard. Species calling abundance was recorded as categories according to Weir & Mossman [30].

For the daytime surveys, we returned to the sites surveyed the previous night and recorded the total number of egg masses per species. Egg masses were identified based on species-specific morphological characteristics. We then conducted 5, 1 m dipnet sweeps to quantify the density of tadpoles, invertebrates and fish. Tadpoles were identified to species and fish and invertebrates were identified to the lowest taxonomic level possible. We also recorded the following environmental variables for each site: 1) surface area – calculated as the maximum length times maximum width of the pool which provides a coarse metric of surface area even though pools were not perfect rectangles; 2) mean depth – which was the mean of 5 depth measurements recorded at random locations in the pool; 3) percent vegetation cover – estimated visually as the amount of the pool surface covered by vegetation; 4) percent canopy cover – calculated as the mean of 4 spherical densiometer measurements taken at the maximum length and maximum width points along the perimeter of the pool; 5) pool substrate – recorded as the presence/absence of these 6 categories: rocks, gravel, cobble, silt, mud, leaf litter. We conducted a principle components analysis on the 6 pool substrate variables to derive a composite pool substrate variable because the pool substrate classes were not mutually exclusive (e.g., a pool could have leaf litter, silt and cobble). The first 2 principle component axes (PC1 and PC2) represented 41% and 25% of the variation, respectively, and summarized different aspects of the pool bottom with silt loading strongly and positively to PC1 and mud and leaf litter loading negatively to PC2. Therefore, we used both PC1 and PC2 in our analyses.

For the statistical analyses, we first identified the environmental variables significantly associated with breeding activity for the entire frog community [31]. We then determined species-specific responses to these significant environmental variables [32]. Because we were interested in determining species-specific habitat preferences, we included in our analyses only pools where we recorded breeding by at least one of the species at least one time. Analyses of the breeding survey data were conducted on calling and oviposition data separately, with evidence of oviposition defined as tadpoles and/or eggs at a site.

We used partial correspondence analysis [31] to determine which environmental variables influenced calling and oviposition site choice for the entire frog community. Partial correspondence analysis performs an ordination of the site data based on species breeding activity which is then constrained by the environmental variables. The analysis considers the effect of each environmental variable on breeding site selection independently of the other variables, which avoids issues of colinearity among variables. We used all environmental variables recorded in the day surveys to constrain the analyses. Due to the high diversity of invertebrate taxa, we grouped taxa into three categories prior to analyses: predatory invertebrates (based on firsthand knowledge of tadpole predation or from the literature; hereafter referred to as predators), non-predatory invertebrates (hereafter referred to as invertebrates) and snails. When snails were present at a site their densities were orders of magnitude higher than other non-predatory invertebrates obscuring any signal of other invertebrates, thus we placed them in their own category. The variables surface area, mean depth, vegetation, canopy cover, fish density, predator density, invertebrate density, and snail density were log transformed and standardized to have a mean of 0 and variance of 1 prior to analyses. We also included tadpole density as an environmental variable in the analysis of the calling data because tadpole density can affect whether a pool is selected for oviposition [33]. Note that the tadpole density variable includes the density of all tadpoles, not just conspecifics at a site.

The partial correspondence analysis indicates which environmental variables are significantly associated with breeding site selection for the whole frog community, but it does not indicate the magnitude or direction of the effect for individual species. We used the lmer function from the lme4 package in R [34,35] to calculate these effects (i.e. habitat preferences) for individual species [36]. We constructed separate models fit to a binomial error distribution for calling and oviposition survey data, where the response variable was presence or absence of calling or oviposition activity. The environmental variables that were identified as statistically significant from the partial correspondence analysis were used as fixed factors in our lmer models, while species was included as a random factor. We allowed species to vary with each environmental variable which permits the calculation of estimates for the effect of that variable on each species’ calling or oviposition activity [37].

### Non-breeding Survey

The goal of non-breeding surveys was to identify which habitats species used during our survey period when they were not breeding. Non-breeding surveys involved visually searching the intervening habitat between breeding sites for frogs and were conducted at the same time as our breeding surveys. Searches were started once we were far enough away from the breeding site (ca. 100 m) as to not include individuals who were actively engaged in breeding. While it is possible that we counted individuals who were migrating to breeding sites in our surveys, they were not actively engaged in breeding activity (e.g. calling) at the time of our encounter.

### Breeding Surveys Outside XTBG

During our preliminary surveys in 2008 and in-depth surveys in 2009 we recorded no breeding activity in the rubber plantation. We wanted to determine whether the absence of breeding in rubber plantations was limited to the plantation at XTBG or occurred in other plantations. Therefore, we surveyed 8 plantations outside of XTBG in 2008, 2009 and 2010. Survey methods were the same as above: we walked a route through the plantation at night to listen for calling and then returned the following day to survey aquatic sites for evidence of oviposition and tadpoles. Because these plantations were not surveyed in the same repeated temporal manner as our other sites, data from these surveys is not included in summaries or analyses and is limited to qualitative support for the absence of breeding in found in the XTBG surveys.

### Rubber Plantation Breeding Habitat Quality

The absence of breeding in the rubber plantation was surprising given that aquatic sites in the other two land-cover types appeared to be readily colonized by breeding frogs including impacted aquatic sites immediately outside (ca. 10 m) the rubber plantation. Most rubber plantations in Xishuangbanna are planted on newly terraced hillsides. The aquatic sites in rubber plantations are rectangular fertilizer pits which are dug by farmers on the terraces in between trees. Pits are filled with fertilizer in March, and it dissipates by the rainy season in June when the pits fill with rain water. Therefore, it was not clear whether frogs were avoiding using these pits due to properties of the pits themselves or because breeding in the rubber plantation was undesirable regardless of pit characteristics. We complemented our surveys with the following three experiments to determine if the rubber plantation fertilizer pits could support frog breeding.

#### i) Laboratory experiment

We conducted an experiment to test whether water from the rubber plantation fertilizer pits is suitable for tadpole growth and development using three common species: *Fejervaryalimnocharis* (Gravenhorst), *Polypedates* *leucomystax* (Gravenhorst), and *Rhacophorusrhodopus* (Liu & Hu). All three species are listed as least concern according to IUCN. *F* *. limnocharis* adults were recorded in the rubber plantation during non-breeding habitat surveys, and were recorded breeding in impacted sites. Neither *P* *. leucomystax* nor *R* *. rhodopus* adults were observed in the rubber plantation, but *P* *. leucomystax* did breed in impacted sites. *F* *. limnocharis* has a larval period of 5-7 weeks and the other two species have larval periods of 6-8 weeks [38]. We collected early-stage (stages 25-30 [39]) tadpoles from ten of our survey pools at XTBG. Tadpoles were randomly assigned to two treatments: “rubber” water which was collected directly from the fertilizer pits and rain water which was collected from a large catchment. Both types of water were filtered through ¼ mm mesh before use in the experiment. We recorded initial weights by haphazardly selecting three individuals of the same species and weighing them together because they were too light to weigh individually. The group of three was then transferred to a unique 2.5 L basin containing either rubber water or rain water. Due to the availability of tadpoles, *F* *. limnocharis* treatments were replicated ten times while *P* *. leucomystax* and *R* *. rhodopus* treatments were replicated three times each for a total of 32 experimental units. Throughout the course of the experiment, tadpoles in both treatments were fed an *ad libitum* diet of *Spirulina* -based fish food, and water was changed every three days. At the end of 14 days, we calculated survival for each basin, and tadpoles were weighed individually and identified to their Gosner developmental stage.

Relative growth rate, survival, and final Gosner developmental stage were used as response variables in statistical analyses. Analyses were conducted on rearing container means within each treatment, and growth rate was log transformed, survival was arcsine square root transformed, and Gosner stage was square root transformed to meet the assumption of normality of variances prior to analysis. We used MANOVA with growth rate, final Gosner stage and survival as the multivariate response variable, and treatment and species as the independent variables. When main treatment effects were significant, we used a Tukey’s honestly significant difference (HSD) test to identify statistically significantly different means.

#### ii) Field experiment

We conducted a field transplant experiment to determine whether the rubber plantation fertilizer pits were suitable for tadpole growth and development. Specifically, we predicted that the pits would be food limited for tadpoles based on their apparent low productivity according to observations that the pools lacked algae and other aquatic organisms that breeding pools contained (J. Behm, *pers. obs.*). Therefore, we raised tadpoles under two treatments: “food added” and “no food added”. We used *F* *. limnocharis* tadpoles only for this experiment due to tadpole availability. We identified 12 fertilizer pits in XTBG’s rubber plantation with consistently high water levels. Pits were on average 50 L (± 11.78 L) in volume (0.6 m x 0.4 m x 0.2 m). Invertebrates were scarce, but the ones that were present were removed from each pit before the pit was covered with 1 mm mesh to permit rain water to enter, to retain tadpoles in the event of flooding, and to prevent predators or frogs from entering the pits. We haphazardly selected nine early-stage *F* *. limnocharis* tadpoles and weighed them as a group, then added the group to a pit. Pits were randomly assigned to a treatment for a total of six replicates per treatment. The food-added pits received a food addition of Spirulina-based fish food for two consecutive days followed by one day without a food addition. The no-food-added pits received no food additions. Water in the pits was closely monitored, and if the water evaporated to less than half full, we refilled it using water from neighboring pits that were not in the experiment. Note that although water levels in the pits fluctuated, this property was not unique to rubber plantation sites as pools in impacted and rainforest sites also experienced water fluctuations due to evaporation and rain. After 14 days, we calculated survival of tadpoles for each pit, and weighed tadpoles individually and identified them to Gosner stage.

We used log-transformed growth rate, arcsine square root-transformed survival and square root-transformed stage as the response variables in statistical analyses. We calculated a fluctuation index for each pit as the total amount of water added to each pit over the course of the experiment. We used MANOVA to analyze these data with growth rate, final Gosner stage and survival as the multivariate response variable, and treatment, pit fluctuation index and pit volume as the independent variables. When main treatment effects were significant, we used a Tukey’s HSD test to identify statistically significantly different means.

#### iii) Pits in other habitats

Rubber plantation fertilizer pits are similar in volume to many of the ephemeral pools where we observed breeding; however, they are narrower and deeper than most commonly used pools in other areas. To determine whether frogs have an aversion to breeding in pools this shape, we dug similar-sized pits at one rainforest and two impacted sites. In May 2010, at each site we dug 10 pits (0.6 m x 0.4 m x 0.2 m) spaced 3-4 m apart as in the rubber plantation. After each large rainfall during the 3-month 2010 breeding season (June–August), we monitored these pits for oviposition.

## Results

### Non-breeding Surveys

**Figure 1:**
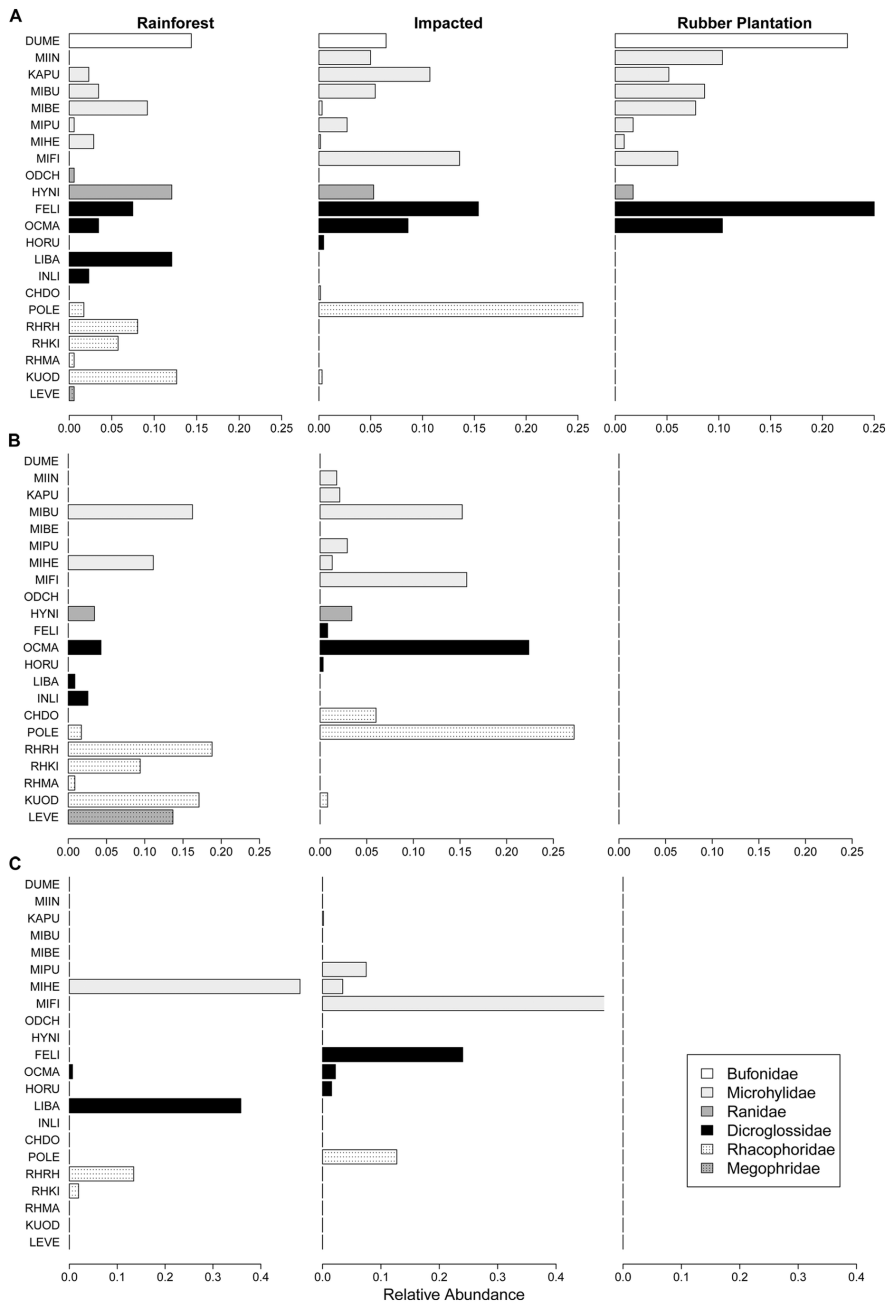
Frog communities at rainforest, impacted and rubber plantation sites from non-breeding and breeding surveys. Relative abundances of the 22 frog species recorded in A) non-breeding surveys, B) calling surveys and C) oviposition surveys in rainforest, impacted and rubber plantation sites. Relative abundances were calculated within each habitat type based on number of individuals encountered (A), male calling abundance (B), and tadpole density (C). Color of bars corresponds to family species belongs to. Species name codes in the order they appear in the figure are: DUME: *Duttaphrynus melanostictus*, MIIN: *Micryletta inornata*, KAPU: *Kaloula pulchra*, MIBU: *Microhyla butleri*, MIBE: *Microhyla berdmorei*, MIPU: *Microhyla pulchra*, MIHE: *Microhyla heymonsii*, MIFI: *Microhyla fissipies*, FELI: *Fejervarya limnocharis*, HORU: *Hoplobatrachus rugulosus*, LIBA: *Limnonectes bannaensis*, INLI: *Ingerana liui*, OCMA: *Occidozyga martensii*, ODCH: *Odorrana chloronota*, HYNI: *Hylarana nigrovittata*, CHDO: *Chiromantis doriae*, POLE: Polypedates leucomystax, RHRH: *Rhacophorus rhodopus*, RHKI: *Rhacophorus kio*, RHMA: *Rhacophorus maximus*, KUOD: *Kurixalus odontotarsus*, LEVE: *Leptolalax ventripunctatus*. See http://www.iucnredlist.org for naming authorities.

During our non-breeding surveys we recorded 22 species across the three land-cover types (Figure 1A). Rainforest sites had the most species (n = 18) followed by impacted sites (n = 15), and rubber plantation sites (n = 11). Because the rainforest had a longer survey route length than the rubber plantation, we wanted to insure that these species richness values were not an artifact of the route lengths. We used a repeated measures ANOVA to analyze the abundance of individuals we encountered per week in each habitat type for the duration of our survey. Despite the rainforest having a longer route length, there was no difference in the abundance of individuals encountered in the rainforest and rubber plantation over the course of the survey (*P* = 0.19, Figure S1). To illustrate how the abundance of individuals is related to species richness in each habitat, we calculated an individual rarefaction curve [40] for the three habitat types using the rarefy function from the vegan library in R [41]. Rarefy estimates the predicted species richness of each habitat as a function of the abundance of individuals in the habitat. These curves show that the high species richness in the rainforest was not due to finding more individuals, but rather there was a more diverse species assemblage in the rainforest (Figure S2).

All 11 species found in the rubber plantation were also found in impacted sites. Nine species were found in all three land-cover types, 7 species were unique to rainforest sites, and 2 species were unique to impacted sites. No species were unique to the rubber plantation sites. Eighteen of the 22 total species we found were from 3 families: Microhylidae (n = 7), Dicroglossidae (n = 5) and Rhacophoridae (n = 6). While Dicroglossids and Microhylids were found in all three land-cover types, Microhylids were predominantly in impacted sites and no Rhacophorids were present in the rubber plantation sites (Figure 1A).

### Breeding Surveys

We repeatedly surveyed 8 pools in the rainforest and 14 pools in impacted sites. While there were no active breeding sites in the rubber plantation our surveys included 300 fertilizer pits, 46 of which consistently held water. Mean distances between pools in the three areas were as follows: rainforest 44.27 ± 55.39 m (range: 7-89 m); impacted 84.20 ± 55.39 m (range: 4-166 m); rubber 13.36 ± 13.34 m (range: 1-52 m). We heard 19 species in total with 12 species calling at rainforest sites, 13 species calling at impacted sites, and no species calling at rubber plantation sites (Figure 1B). The rainforest and impacted sites shared 6 species, 6 species were unique to the rainforest and 7 species were unique to impacted sites.

We recorded oviposition for 11 species with 5 species ovipositing in rainforest sites, 8 species ovipositing in impacted sites and no species ovipositing in rubber plantation sites (Figure 1C). The rainforest and impacted areas shared 2 species, and there were 3 species unique to rainforest and 6 species unique to impacted sites. The taxonomic pattern for breeding was similar to the pattern exhibited in the non-breeding surveys. Species that were unique to rainforest sites were mostly Rhacophorids and species that were unique to impacted sites were mostly Microhylids. In our surveys of eight rubber plantations outside of XTBG, we recorded no incidents of calling or oviposition.

**Figure 2:**
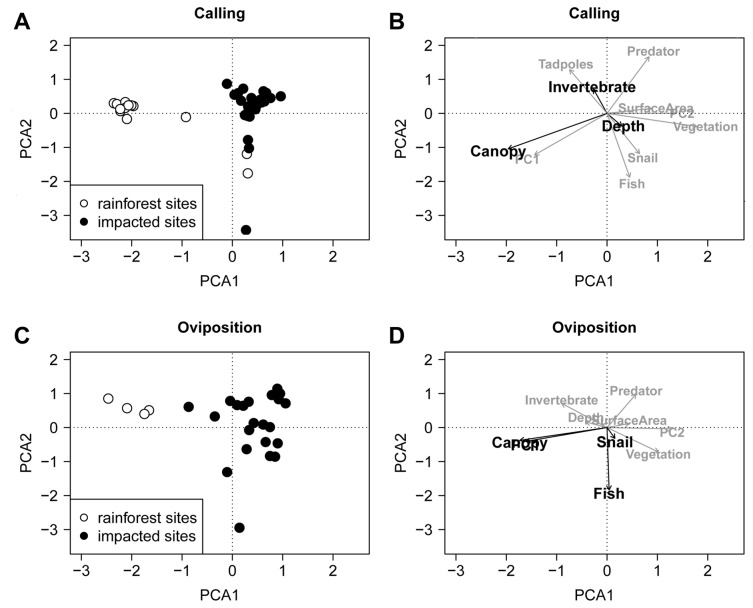
Composition of breeding sites according to environmental variables. Plots from the correspondence analysis of breeding survey sites showing composition of A) survey sites and B) associated 11 environmental variables from calling surveys. Plots C) and D) show the same information for oviposition surveys based on 10 environmental variables. Significant environmental variables according to the partial correspondence analysis are in bold in plots B and D. PC1 (silt) and PC2 (mud and leaf litter) refer to pool substrate.

In our daytime breeding site surveys, we encountered 4 fish taxa, 9 predatory invertebrate taxa and 10 non-predatory invertebrate taxa (Table 1). Calling was significantly influenced by pool depth, percent canopy cover and invertebrate density while oviposition was influenced by PC1 (silt), canopy cover, fish and snail density (Table 2). The sites clustered into three groups according to environmental variables (Figure 2A, C). For calling activity, the majority of sites were positively associated with invertebrates and negatively associated with depth, while rainforest sites were positively associated with high percent canopy cover (Figure 2B). This was more or less the same pattern for oviposition sites with fish and snail density affecting sites in the same direction as depth, and PC1 in the same direction as percent canopy cover (Figure 2D).

**Table 1:** Fish, predatory invertebrates, and non-predatory invertebrates identified in breeding site surveys.

**Fish**	**Predatory Invertebrates**	**Non Predatory Invertebrates**
*Oreochromis* spp.	Epiprocta larvae	Gastropod (snail)
*Gambusia* spp.	Zygoptera larvae	Caridea (shrimp)
*Carassius* spp.	Notonectidae	Brachyura (crab)
Small darter-like *Percidae*	Belastomatidae	Culicidae larvae
	Aquatic Araneae	Chrionomid larvae
	Dytiscid larvae	Hirudinea
	Dytiscid adults	Oligochaeta
	Gerridae adults	Coleopteran larvae
	Ranatra adults	Ephemeropteran larvae
		Trichopteran larvae

**Table 2:** Percent variation explained, *P*-value, and coordinates for the first two axes (PCA1 and PCA2) for environmental variables from partial correspondence analysis.

	**Calling**				**Oviposition**			
	**Variation explained**	***P-***	**PCA**	**PCA**	**Variation explained**	***P-***	**PCA**	**PCA**
**Variable**	**(%)**	**value**	**1**	**2**	**(%)**	**value**	**1**	**2**
Surface Area	1.51	0.11	0.34	0.06	2.01	0.10	0.21	-0.06
Depth	2.80	0.01	0.11	-0.14	1.96	0.11	-0.20	-0.08
Vegetation	0.75	0.67	0.69	-0.15	0.53	0.90	0.49	0.35
Silt (PC1)	1.44	0.12	-0.56	-0.47	3.42	0.01	-0.78	0.20
Mud and leaf litter (PC2)	1.61	0.08	0.52	0.00	2.00	0.09	0.62	0.02
Canopy Cover	4.00	0.00	-0.76	-0.40	3.80	0.01	-0.83	0.18
Fish	1.31	0.20	0.17	-0.72	3.25	0.01	0.02	0.88
Predators	1.36	0.16	0.32	0.64	0.64	0.83	0.28	-0.47
Invertebrates	2.17	0.01	-0.10	0.28	1.01	0.58	-0.43	-0.34
Snails	1.26	0.22	0.25	-0.46	2.52	0.04	0.08	0.15
Tadpoles	1.68	0.05	-0.29	0.50	-	-	-	-

We used a multilevel linear model to calculate effect sizes for each species for the significant environmental variables that affected calling and oviposition (Table 3). The environmental variable with the highest effect sizes was percent canopy cover. On average, species that bred only in the rainforest had positive effect sizes for percent canopy cover while species that bred in impacted sites had negative effect sizes for percent canopy cover (Table 3). Overall, the mean percent canopy cover across rainforest sites was high (mean ± standard deviation = 71.2 ± 26.5%), and impacted sites it was low (28.8 ± 24.1%). Although no species bred in the rubber plantation, percent canopy cover above rubber plantation pools was high (72.3 ± 11.6%).

**Table 3:** Multilevel model effect size estimates (Est.) and standard errors (S.E.) for each frog species with respect to environmental variables identified in partial correspondence analyses to significantly impact calling and oviposition.

	**Calling**						**Oviposition**							
**Species**	**Depth**		**Canopy**		**Invertebrate**		**PC1**		**Canopy**		**Fish**		**Snail**	
	Est.	S.E.	Est.	S.E.	Est.	S.E.	Est.	S.E.	Est.	S.E.	Est.	S.E.	Est.	S.E.
**Breeds in Rainforest**														
*Limnonectesbannaensis*	0.09	0.01	0.84	0.01	-0.03	0.01	0.11	0.04	2.42	0.03	0.03	0.03	-0.20	0.03
*Rhacophorusrhodopus*	0.21	0.02	3.66	0.03	0.16	0.03	0.12	0.02	1.47	0.03	-0.08	0.02	-0.05	0.02
*Rhacophorus kio*	0.13	0.02	0.29	0.03	0.07	0.03	0.42	0.01	0.44	0.01	-0.29	0.01	0.11	0.01
*Rhacophorus* *maximus*	0.06	0.01	0.83	0.01	0.12	0.01	-	-	-	-	-	-	-	-
*Leptolalax*	0.05	0.02	3.98	0.03	-0.09	0.03	-	-	-	-	-	-	-	-
*ventripunctatus*														
**Breeds in Impacted**														
*Micryletta* *inornata*	0.13	0.02	0.08	0.01	0.05	0.03	-		-		-		-	
*Kaloula* *pulchra*	-0.20	0.01	-0.24	0.02	0.30	0.02	-0.09	0.02	-0.13	0.02	-0.45	0.02	0.15	0.02
*Microhyla* *pulchra*	0.39	0.01	-1.89	0.02	0.05	0.02	0.28	0.03	-1.72	0.03	-0.39	0.02	0.17	0.02
*Microhylafissipes*	-0.18	0.03	-1.32	0.04	-0.09	0.04	-0.87	0.05	-0.71	0.05	-0.03	0.04	-0.08	0.05
*Fejervaryalimnocharis*	-0.30	0.01	-1.26	0.02	-0.05	0.02	-0.17	0.05	-1.24	0.05	-0.19	0.04	-0.29	0.04
*Hoplobatrachus*	-0.07	0.01	-0.59	0.01	0.08	0.01	0.09	0.02	-0.85	0.02	-0.40	0.02	0.10	0.02
*rugulosus*														
*Chiromantus* *doriae*	0.01	0.02	-2.31	0.03	-0.23	0.03	-	-	-	-	-	-	-	-
*Polypedates*	-0.43	0.03	-0.80	0.05	0.08	0.05	-0.11	0.06	-0.61	0.06	3.44	0.05	-0.24	0.05
*leucomystax*														
**Breeds in Both**														
*Microhyla* *butleri*	-0.01	0.02	-1.95	0.03	-0.01	0.03	0.31	0.03	0.02	0.05	-0.61	0.03	0.28	0.04
*Microhylaheymonsi*	-0.01	0.02	0.28	0.03	-0.06	0.03	0.12	0.05	-0.41	0.05	0.52	0.04	-0.51	0.04
*Occidozygamartensii*	-0.32	0.03	-0.93	0.05	0.14	0.05	-0.59	0.04	0.53	0.03	2.96	0.03	0.22	0.03
*Hylarananigrovitata*	0.45	0.02	0.78	0.03	-0.40	0.03	-		-		-		-	
*Kurixalusodontotarsus*	0.13	0.02	0.08	0.01	0.05	0.03	-		-		-		-	

Breeding habitat preference was categorized based on oviposition habitat choice, or calling habitat choice in the absence of recorded oviposition.

Note: standard errors were estimated through a fixed effect-only model.

### Rubber Plantation Breeding Habitat Quality

#### i) Laboratory Experiment

**Figure 3:**
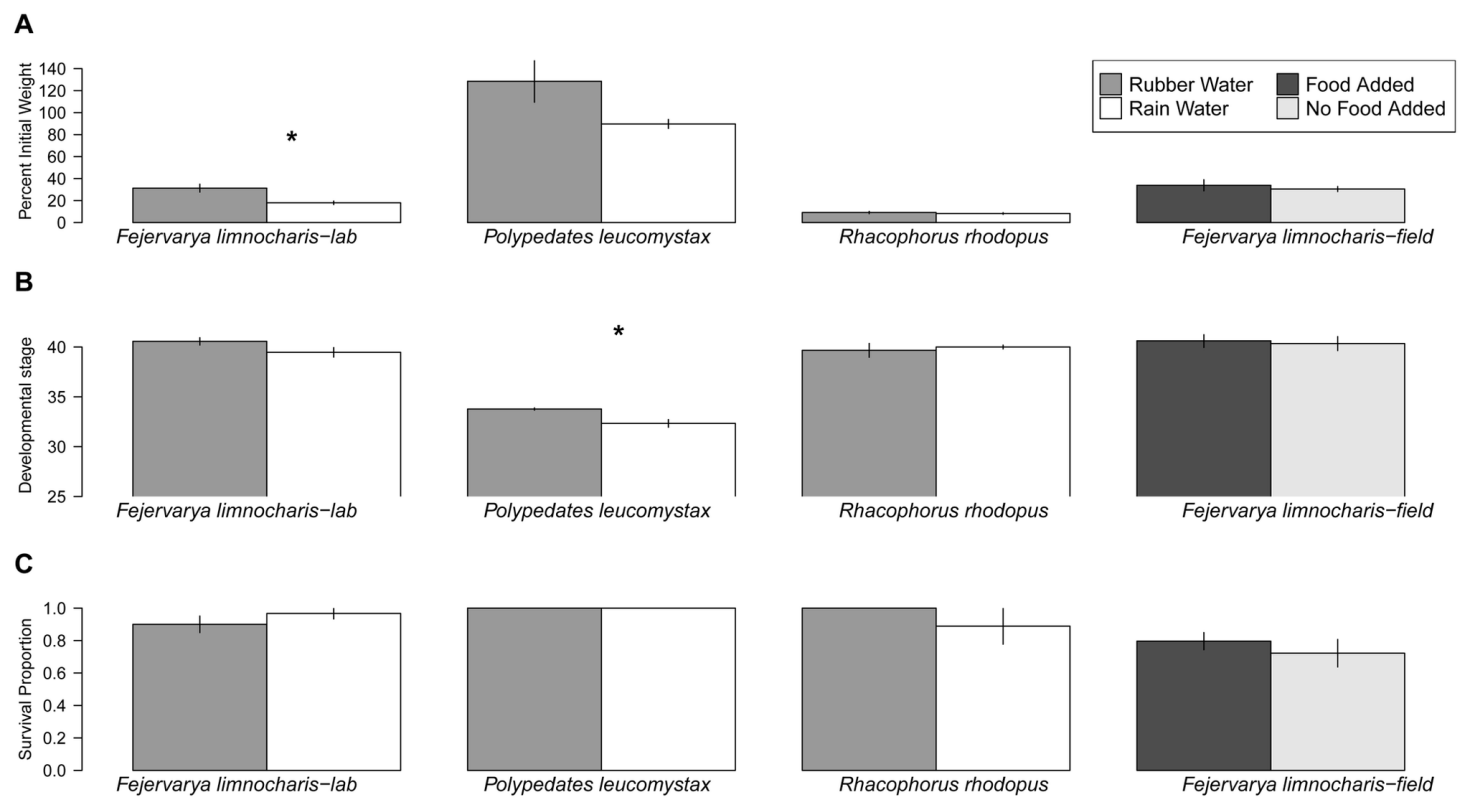
Results from laboratory and rubber plantation field transplant experiments. Means (± SE) from laboratory (rubber water vs. rain water) and field experiments (food added vs. no food added) using three tadpole species. A) percent of initial weight grown per day; B) final developmental stage; C) proportion surviving. Asterisks indicate significant differences between treatments within a species according to Tukey post hoc tests that account for multiple comparisons.

When three species of tadpoles were grown in rubber water versus rain water, there was an overall significant effect of treatment (Pillai’s trace = 0.44, *F*_1,26_ = 6.35, *P* < 0.01), species (Pillai’s trace = 1.45, *F*_2,26_ = 21.72, *P* < 0.01) and no significant treatment by species interaction (Pillai’s trace = 0.27, *F*_2,26_ = 1.31, *P* = 0.27). Tadpoles had higher growth (*F*_1,26_ = 12.30, *P* < 0.01; Figure 3A) and higher development in the rubber water treatments (*F*_1,26_ = 4.82, *P* < 0.05; Figure 3B) compared to rain water, but survival was equal in both treatments (*F*_1,26_ = 0.22, *P* = 0.64; Figure 3C). *F* *. limnocharis* had higher growth in rubber water than rain water (*P* < 0.05, TukeyHSD), and *P* *. leucomystax* had higher development in rubber water than rain water (*P* < 0.05, TukeyHSD). There were significant species effects for growth (*F*_2,26_ = 25.29, *P* < 0.01; Figure 3A) due to *P* *. leucomystax* having higher growth than *F* *. limnocharis* (*P* < 0.01, TukeyHSD), and *F* *. limnocharis* having higher growth than *R* *. rhodopus* (*P* < 0.01, TukeyHSD). Significant species effects for developmental stage (*F*_2,26_ = 26.19, *P* < 0.01; Figure 3B) were caused by *P* *. leucomystax* having lower development than the other two species (*P* < 0.01 TukeyHSD).

#### ii) Field Experiment

There were no significant effects of food treatment (Pillai’s trace = 0.03, *F*_1,8_ = 0.06, *P* = 0.98), pit volume (Pillai’s trace = 0.22, *F*_1,8_ = 0.58, *P* = 0.65) or pit fluctuation (Pillai’s trace = 0.35, *F*_1,8_ = 1.07, *P* = 0.43) on *F* *. limnocharis* growth, development or survival. Tadpoles displayed equally high growth (*F*_1,8_ = 0.13, *P* = 0.73; Figure 3A), development (*F*_1,8_ = 0.08, *P* = 0.78; Figure 3B), and survival (*F*_1,8_ = 0.21, *P* = 0.66; Figure 3C) in the food added and no food added treatments. *F* *. limnocharis* tadpoles grown in the lab and field had comparable growth rates, and development rates, while tadpoles grown in the lab had slightly higher survival (Figure 3).

#### iii) Pits in other habitats

We recorded oviposition repeatedly throughout the breeding season in both rainforest and impacted land-cover types where we dug pits similar in shape to rubber plantation fertilizer pits. In the rainforest, we recorded oviposition by *Limnonectesbannaensis* (Ye, Fei, Xie & Jiang) and *R* *. rhodopus* . In the impacted sites, we recorded oviposition by *Microhylafissipes* (Boulenger)*,* *M* *. butleri* (Boulenger)*,* *M* *. heymonsi* (Vogt)*,* *Micryletta* *inornata* (Boulenger)*,* *F* *. limnocharis* , and *P* *. leucomystax* *.*

## Discussion

By integrating results from breeding and non-breeding habitat surveys, we showed that the frog species in Xishuangbanna have distinct habitat preferences. One group indicated a preference for only rainforest habitat for both breeding and non-breeding activities according to our surveys. The conversion of rainforest to rubber plantations represented a complete loss of habitat for these species. In addition, no species completed their life cycle solely in the rubber plantation because no species bred there during our study period.

### Rubber plantation breeding habitat quality experiments

In other systems, frogs avoid breeding in sites that are detrimental to tadpoles [42–44]. Pesticides and fertilizer have been shown to have significant impacts on tadpole growth, development and survival [45–48]. Due to the high use of pesticides in rubber plantations and fertilizer directly in the pits, we conducted a set of experiments to determine whether the pools were detrimental to tadpoles, which could possibly explain why the pools were avoided for breeding. The lab experiment showed that water from the rubber plantation pools was suitable for the growth, development and survival of three tadpole species. In fact, *F* *. limnocharis* and *P* *. leucomystax* tadpoles performed better in the rubber water treatment versus the rainwater treatment. This was likely due to small particles from the rubber water passing through our filter, possibly providing an additional food source for the tadpoles. Nonetheless, the rubber water was not detrimental to the tadpoles. The field transplant experiment supported the lab experiment results: tadpoles grew, developed and survived in the rubber plantation fertilizer pits. Further, there was no effect of our food addition treatment, indicating that the pits provided abundant food resources for the tadpoles. Finally, the shape of the pits did not appear to be a deterrent either: frogs readily oviposited in pit-shaped pools that we dug in rainforest and impacted areas.

### Why are no frogs breeding in the rubber plantation?

**Figure 4:**
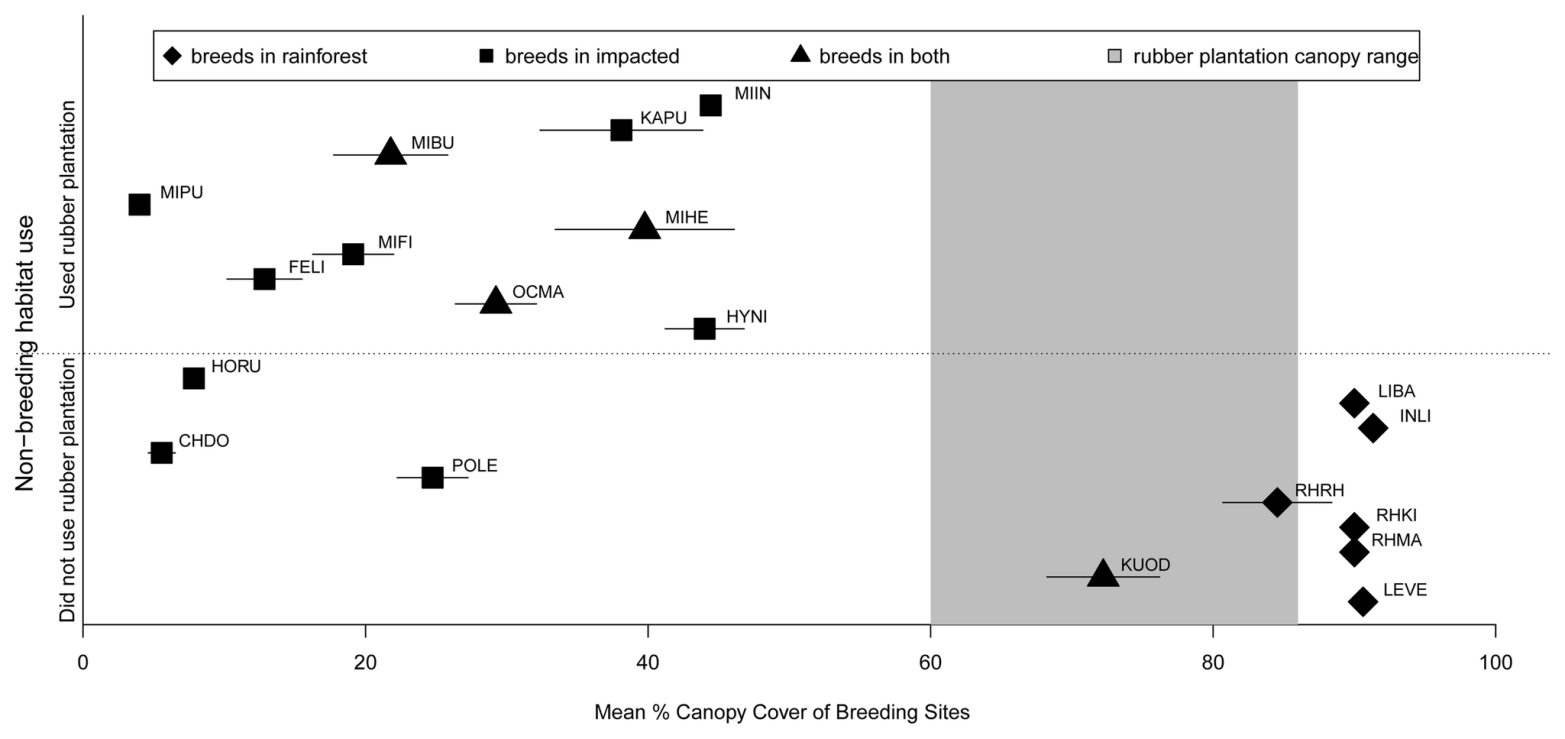
Integrated breeding and non-breeding habitat preferences. Symbols indicate the mean percent canopy cover (± SE) of breeding sites used by each species according to their preferred breeding habitat (rainforest, impacted or both rainforest and impacted). Species in the top panel used the rubber plantation as non-breeding habitat, and species in the bottom panel did not. Species codes next to each symbol are the same as in Figure 1.

Our experimental results suggest that the absence of breeding in the rubber plantation was likely not due to intrinsic properties of the plantation pools themselves; however, one crucial question still remains: Why are no frogs breeding in the rubber plantation? Our breeding survey analyses showed that canopy cover was the strongest environmental factor influencing breeding site selection, with the species in our survey exhibiting preferences for both high and low canopy cover. This is puzzling given that rubber plantation sites also have high canopy cover but were avoided, until the relationship between breeding and non-breeding habitat preferences are considered. We summarized these relationships between non-breeding habitat use and breeding habitat canopy cover preferences in Figure 4. The species that bred in high canopy cover sites were the species that only used the rainforest as non-breeding habitat (Figure 4). Their life cycle is completed entirely within the rainforest. Rubber plantation sites are likely suitable for the growth, development and survival of their tadpoles, yet other properties of the rubber plantation likely make it unsuitable non-breeding habitat so it is avoided entirely. In contrast, the species that did use the rubber plantation as non-breeding habitat left the rubber plantation to breed in low canopy cover sites (Figure 4). We suggest that rubber plantations are an intermediate habitat type that ‘slips through the cracks’ of the species’ habitat preferences and this is why species avoid breeding there.

### Breeding habitat preferences

Canopy cover can influence the conditions of a pool affecting temperature, food quality and the biotic community [49–52], and is a strong driver in breeding site selection for North American frog species as well [42,53]. A preference for low canopy cover sites is generally attributed to tadpoles of that species having higher growth rates in low versus high canopy cover pools [50,52]. Although these tadpoles can survive in high canopy cover pools, their growth (and likely fitness) is higher in low canopy cover pools, thus high canopy cover pools are avoided [50]. We suspect this mechanism may be at work in our system. *F* *. limnocharis* adults were abundant in rubber plantations and their tadpoles had high survival in rubber plantation pools during our field transplant experiment, yet they bred in low canopy cover pools and not the rubber plantation. *F* *. limnocharis* tadpoles grown at comparable densities in containers in low canopy cover conditions from a separate study (J. Behm *unpublished data*) had slightly higher growth rates than those from our transplant experiment (F_1,15_ = 5.04, *P* = 0.04). While this result is by no means conclusive, it does suggest that sites outside of the rubber plantation may provide higher growth rates for *F* *. limnocharis* , and may be why rubber plantation sites are avoided. Identifying growth, development and survival rates of multiple species in the field at impacted, rubber plantation and rainforest sites would be an interesting avenue for future work.

In addition to canopy cover, invertebrate density and pool depth influenced calling site selection while silt substrate (PC1), snail density and fish density affected oviposition site selection. While it appears that calling and oviposition site selection are influenced by different variables, the variables have similar associations with the axes and are indicative of the same types of habitats. For example, fish were more common in deeper pools. When fish were present, invertebrates were scarce and snail densities were high – likely because fish eat invertebrates and snails are protected from fish predation by their shells. Thus the variables invertebrate density, depth, snail density and fish density are likely summarizing the same type of habitats: deep pools with fish and shallow pools without fish. The majority of species in our survey avoided pools with fish, as has been shown in other systems [33,54,55]. We strongly recommend limiting fish introductions to pools within Xishuangbanna to protect the frog species.

Three species, *Microhylaheymonsi* *,* *M* *. butleri* , and *Occidozygamartensii* (Peters), used the rubber plantation as non-breeding habitat and also oviposited in both high and low canopy sites. Based on these preferences, we would expect that these are three good candidate species for breeding in the rubber plantation. The fact that we did not record them breeding in the rubber plantation indicates that there may be additional environmental features frogs are avoiding that we did not measure. Finding the aquatic sites in the rubber plantation is likely not an issue because ephemeral pool-breeding species are generally good at finding new aquatic sites [26–28] and we found that to be true in our system as well. For example, we observed *O* *. martensii* and *F* *. limnocharis* adults swimming in rubber plantation aquatic sites on several occasions.

### Non-breeding habitat preferences

Non-breeding habitat is selected based on microhabitat characteristics which prevent desiccation, facilitate movement, and provide food and safety [19,56–58]. The rubber plantation was inferior non-breeding habitat compared to the other two land-cover types with respect to species richness and composition. The species missing from rubber plantation were phylogenetically non-random: Rhacophorids were absent, while only some Dicroglossids and all Microhylids were present. The loss of an entire evolutionary lineage in rubber plantations represents a greater loss of biodiversity than if the same number of species were lost randomly across multiple families [59]. Because closely related species generally share similar habitat needs, ecological characteristics of the rubber plantation may be responsible for this phylogenetic pattern. Amphibian richness in cacao plantations in Sulawesi is influenced by microclimates provided by plantation features such as logs and leaf litter [19]. Rubber plantations lack understory vegetation and are likely more dry than the other two land cover types [60]. Microhylids are leaf litter frogs that are likely resistant to desiccation and can possibly better tolerate conditions in the rubber plantation. Rhacophorids are treefrogs, and in Madagascar arboreal species are also highly sensitive to habitat modification [61]. Some Rhacophorids in our survey are highly arboreal (e.g. *Rhacophorus* genus) while others are less so, therefore, there are likely different mechanisms responsible for the lack of Rhacophorid species in rubber plantation.

Most amphibians are thought to exist in a metapopulation structure [62] thus dispersal between populations, mostly in the juvenile stage [2,63], is incredibly important to maintaining amphibian population persistence [64]. In our system, all Rhacophorid species avoided the rubber plantation. This may indicate that rubber plantations are dispersal barriers for these species, but this should be confirmed by estimating genetic isolation for these populations through non-destructive means. In addition, adults migrate between non-breeding habitat and breeding habitat during the breeding season, with migration distances on average being significantly less than dispersal distances [65]. If rubber plantations are barriers to migration, populations may decline [23,25].

Although the rubber plantation had the lowest species richness with no unique species, 11 species used it as non-breeding habitat indicating it may have some useful microhabitat characteristics. Rubber plantations may be a high quality habitat for some species if they provide valuable or unique resources. For example, the invasive earthworm, *Pontoscolexcorethrurus* , is highly abundant in rubber plantations [66], and we observed *F* *. limnocharis* juveniles eating earthworms (J. Behm pers*. obs.*). Alternatively, it is possible that rubber plantations could be a sink habitat if, for example, food resources or pesticide levels caused low fitness for individuals in the rubber plantation [67]. Assessing the quality of non-breeding habitat provided by rubber plantations should be an area for future research.

### Frog conservation in Xishuangbanna

Our study was one of the first assessments of how the massive conversion of rainforests into rubber plantations is affecting native biodiversity in Xishuangbanna. Our study was an intensive survey of the frog community in one plantation adjacent to one rainforest patch – how much can we generalize from this study to the rest of Xishuangbanna and other commercial rubber growing regions in Southeast Asia? Our survey was likely a best-case scenario for finding high frog diversity in rubber plantations in Xishuangbanna as XTBG’s rubber plantation is relatively old, is not as intensively managed as other plantations, and is adjacent to a relatively large remnant rainforest patch which likely acts as a source for frog species. Although it is possible that rubber plantations adjacent to larger rainforest patches could have higher frog diversity. In terms of breeding ecology, if plantations in the region have similar characteristics to the one at XTBG, and the frogs have similar habitat preferences to the ones at XTBG, our results may be applicable. In their current state, upland rubber plantations adjacent to lowland impacted areas may provide sufficient habitat resources for the entire life cycle for some frog species. Like many developing areas, urban development in Xishuangbanna is non-random with respect to topography, and lowland areas generally are developed first [68]. The continuation of this trend will further eliminate breeding sites for species that breed in these lowland impacted sites. At this point, spatially extensive surveys of rubber plantations across the region are necessary to confidently predict how frog populations will fare in the future.

It may be possible to modify rubber plantations to make them higher quality non-breeding habitat for more species in order to satisfy the often antagonistic goals of providing a livelihood for local residents while enhancing biodiversity. Perhaps increasing the amount of understory vegetation in rubber plantations would provide necessary microclimates that would protect against desiccation for slightly more sensitive species. At this point our only suggestion for modifying rubber plantations to make them more attractive for frog breeding would be to reduce canopy cover. The canopy cover in the rubber plantation at XTBG was near the higher end of the range of rubber plantations we surveyed; however, we found no frogs breeding in rubber plantations with lower canopy cover outside XTBG. It is important to note that these plantations with lower canopy cover still had much higher canopy cover than most of the impacted sites in our survey.

In conclusion, we agree with Gibson et al. [5]: there is no substitute for primary forests for biodiversity. Amphibians across the globe are deeply imperiled [69] and southeast Asian species are no exception [70]. There is likely no way to modify rubber plantations to make them attractive to the species in our survey that were solely reliant on the rainforest as habitat. In order to conserve the unique frog community in Xishuangbanna, remnant forest patches must be preserved and primary forest restored.

## Supporting Information

Figure S1 **Abundance of individuals encountered in each habitat per week.** We encountered more individuals in impacted compared to rubber plantation (*P* < 0.001) and rainforest (*P* < 0.001) areas, while there was no difference in the number of individuals we encountered in rubber plantation and rainforest areas (*P* = 0.19).
(TIF)

Figure S2 **Individual rarefaction curves.** Rarefaction curves for each habitat type generated by the rarefy function in the vegan library in R. Rarefy calculates the expected species richness for each of the three habitat types given a random subsample of a number of individuals from that community. This shows that for a random number of individuals selected from each habitat, chances are higher that they will include more species if the sample is taken from the rainforest community. Vertical lines represent one standard error of the mean.
(TIF)
