# Supplementary figures and images for: Slipping through the Cracks: Rubber Plantation Is Unsuitable Breeding Habitat for Frogs in Xishuangbanna, China
Source: PLoS One. 2013 Sep 10;8(9):e73688. doi: 10.1371/journal.pone.0073688 (PMC3769397; doi:10.1371/journal.pone.0073688)

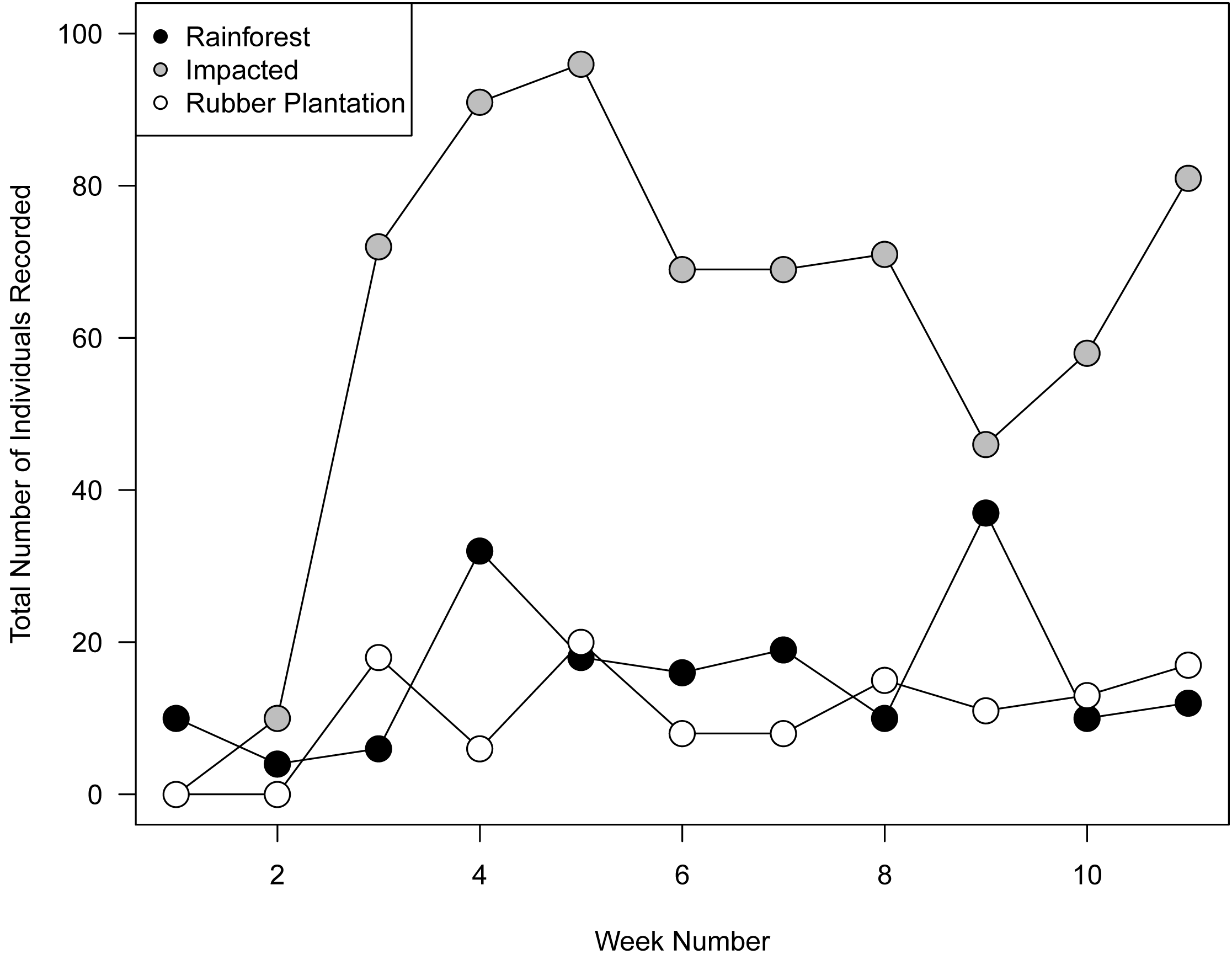

Supplement: Figure S1 — Abundance of individuals encountered in each habitat per week. We encountered more individuals in impacted compared to rubber plantation (P < 0.001) and rainforest (P < 0.001) areas, while there was no difference in the number of individuals we encountered in rubber plantation and rainforest areas (P = 0.19). (TIF) [file pone.0073688.s001.tif]

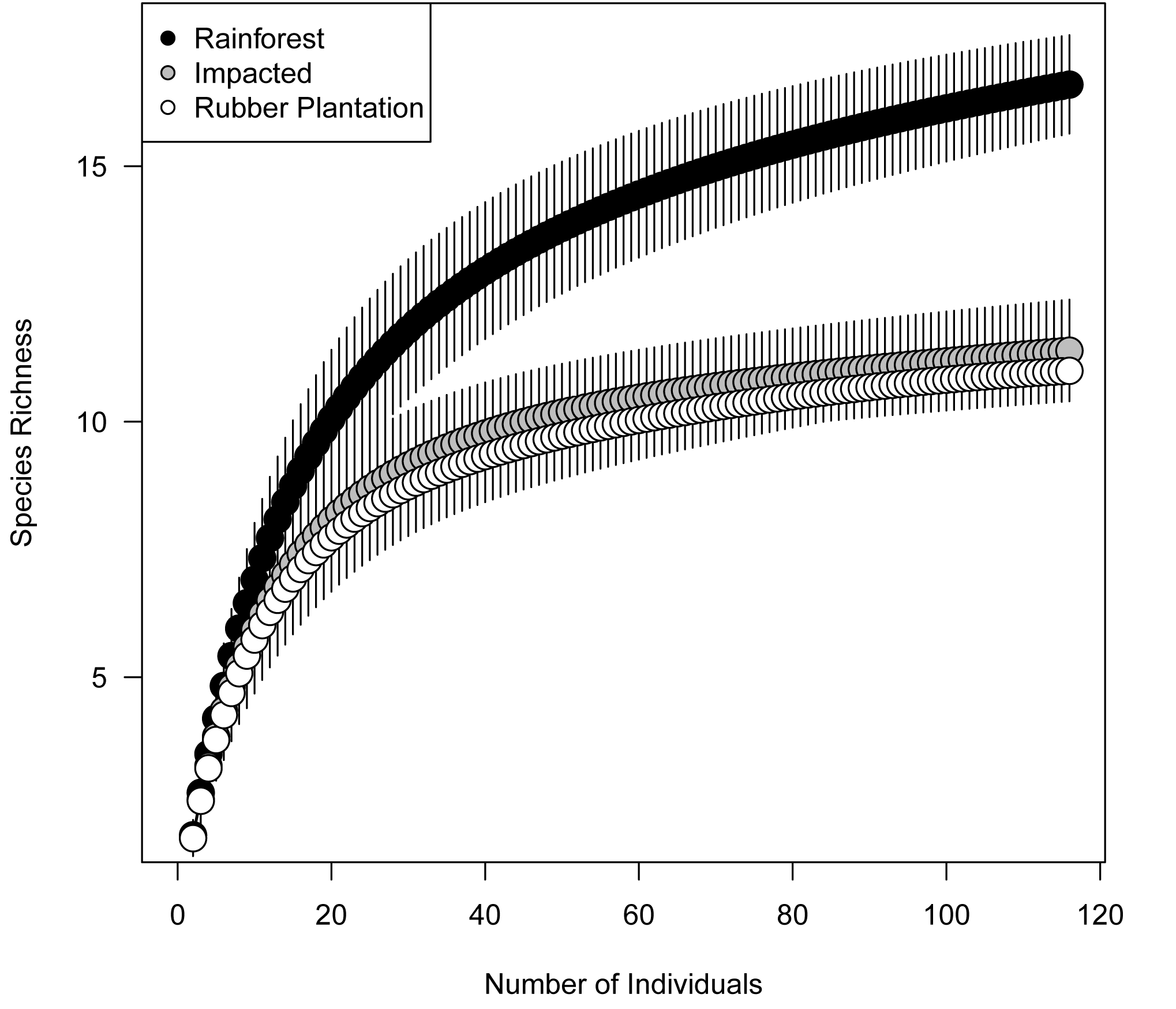

Supplement: Figure S2 — Individual rarefaction curves. Rarefaction curves for each habitat type generated by the rarefy function in the vegan library in R. Rarefy calculates the expected species richness for each of the three habitat types given a random subsample of a number of individuals from that community. This shows that for a random number of individuals selected from each habitat, chances are higher that they will include more species if the sample is taken from the rainforest community. Vertical lines represent one standard error of the mean. (TIF) [file pone.0073688.s002.tif]
